# Exosomes derived from adipose-derived stem cells alleviate cigarette smoke-induced lung inflammation and injury by inhibiting alveolar macrophages pyroptosis

**DOI:** 10.1186/s12931-022-01926-w

**Published:** 2022-01-11

**Authors:** Zhixing Zhu, Xihua Lian, Xiaoshan Su, Weijing Wu, Yiming Zeng, Xiaoyang Chen

**Affiliations:** 1 Department of Pulmonary and Critical Care Medicine, The Second Affiliated Hospital of Fujian Medical University, Respirology Medicine Centre of Fujian Province, 34 Zhongshanbei Road, Licheng District, Quanzhou, China; 2 Department of Ultrasound Medicine, The Second Affiliated Hospital of Fujian Medical University, 34 Zhongshanbei Road, Licheng District, Quanzhou, China

**Keywords:** Adipose-derived stem cells, Exosomes, Chronic obstructive pulmonary disease, Inflammation, Mucus secretion, Alveolar macrophages, Pyroptosis

## Abstract

**Background:**

Chronic obstructive pulmonary disease (COPD) is a frequently encountered disease condition in clinical practice mainly caused by cigarette smoke (CS). The aim of this study was to investigate the protective roles of human adipose-derived stem cells-derived exosomes (ADSCs-Exo) in CS-induced lung inflammation and injury and explore the underlying mechanism by discovering the effects of ADSCs-Exo on alveolar macrophages (AMs) pyroptosis.

**Methods:**

ADSCs were isolated from human adipose tissues harvested from three healthy donors, and then ADSCs-Exo were isolated. In vivo, 24 age-matched male C57BL/6 mice were exposed to CS for 4 weeks, followed by intratracheal administration of ADSCs-Exo or phosphate buffered saline. In vitro, MH-S cells, derived from mouse AMs, were stimulated by 2% CS extract (CSE) for 24 h, followed by the treatment of ADSCs-Exo or phosphate buffered saline. Pulmonary inflammation was analyzed by detecting pro-inflammatory cells and mediators in the bronchoalveolar lavage fluid. Lung histology was assessed by hematoxylin and eosin staining. Mucus production was determined by Alcian blue-periodic acid-Schiff staining. The profile of AMs pyroptosis was evaluated by detecting the levels of pyroptosis-indicated proteins. The inflammatory response in AMs and the phagocytic activity of AMs were also investigated.

**Results:**

In mice exposed to CS, the levels of pro-inflammatory cells and mediators were significantly increased, mucus production was markedly increased and lung architecture was obviously disrupted. AMs pyroptosis was elevated and AMs phagocytosis was inhibited. However, the administration of ADSCs-Exo greatly reversed these alterations caused by CS exposure. Consistently, in MH-S cells with CSE-induced properties modelling those found in COPD, the cellular inflammatory response was elevated, the pyroptotic activity was upregulated while the phagocytosis was decreased. Nonetheless, these abnormalities were remarkably alleviated by the treatment of ADSCs-Exo.

**Conclusions:**

ADSCs-Exo effectively attenuate CS-induced airway mucus overproduction, lung inflammation and injury by inhibiting AMs pyroptosis. Therefore, hADSCs-Exo may be a promising cell-free therapeutic candidate for CS-induced lung inflammation and injury.

**Supplementary Information:**

The online version contains supplementary material available at 10.1186/s12931-022-01926-w.

## Background

Chronic obstructive pulmonary disease (COPD) is defined as a preventable and treatable disease that is usually progressive and associated with an enhanced chronic inflammatory response in the airways and the lung to noxious particles or gases [1]. COPD is a global health epidemic predominantly caused by cigarette smoke (CS), and it is responsible for more than 3 million deaths worldwide every year and the third leading cause of death in the world. In addition, it is also a leading cause of chronic morbidity [2]. Pulmonary architectural disruption induced by chronic airway inflammation triggered by CS is arguably one of the most intractable problems of COPD, resulting in irreversible loss of lung function [3, 4]. Although great strides have been made in developing the treatment options for COPD, unfortunately, the currently available treatments, including pharmacotherapy, pulmonary rehabilitation and oxygen therapy as well as lung-volume reduction, have limited efficacy and do not significantly attenuate lung architectural destruction, prevent disease progression and reduce disease mortality [5, 6].

Mesenchymal stem cells (MSCs) have gained wide interest in the treatment of diseases causing pulmonary architectural disruption due to their potential ability to repair and regenerate lung tissue after injury [7, 8]. MSCs originate from a wide range of tissues, including the adipose tissue, bone marrow, umbilical cord and amniotic fluid [9, 10]. Among these tissues, the adipose tissue represents an attractive source of MSCs and adipose-derived MSCs (ADSCs) seem to be the most promising cell candidate in cell-based therapy and tissue regeneration [10, 11]. Although the potential ability of ADSCs in attenuating COPD-related lung injury and repairing pulmonary structure abnormalities has been initially studied, it is poorly elucidated compared with that of other MSCs [12, 13]. Moreover, most of the studies have mainly focused on elastase-induced COPD [14–16], thus, the potential therapeutic effects of ADSCs on CS-induced lung inflammation and injury and COPD together with the underlying mechanisms remain to be explored [16, 17].

Emerging evidence suggested that the MSCs-derived exosomes (Exo)-mediated paracrine effect is a major mechanism of MSCs-based therapy [18]. Exo are small membrane vesicles ranging in size from 40 to 160 nm and are secreted by many cells [19, 20]. These membranous vesicles can transfer the cargo of the cell of origin, thereby conveying information to the recipient cells. Considering the role of Exo in intercellular communication, the effects of MSCs-derived Exo in many pathological conditions, including pulmonary diseases, are being explored [20, 21]. Although a previous study has initially shown that ADSCs-derived Exo (ADSCs-Exo) reversed emphysematous lung disruption caused by elastase [22], little is known about the effects of ADSCs-Exo on CS-induced pulmonary injury and the underlying mechanisms.

Alveolar macrophages (AMs) are the most abundant immunocytes in the lung, and they play critical roles in host defence, tissue repair, and lung homeostasis [23]. AMs dysfunction plays a significant role in the pathogenesis of COPD [24, 25]. Recently, accumulating evidence indicates that various death pathways of AMs are largely involved in the development and progression of pulmonary inflammation, which greatly contributes to the induction and exacerbation of various airway diseases [26, 27]. Pyroptosis is a newly documented inflammatory programmed cell death pathway induced by inflammatory assault and activated by several inflammatory caspases [28]. Cumulative studies have shown that pyroptosis is fundamentally central to the pathogenesis of many chronic pulmonary disorders, including CS-induced lung inflammation and injury and COPD. Importantly, pyroptosis inhibition has shown promise as a therapeutic target for COPD [29]. However, no attempts have been made to study the profile of AMs pyroptosis that occurs in CS-induced lung inflammation and injury.

Herein, we hypothesized that CS exposure can elevate the level of AMs pyroptosis and ADSCs-Exo can attenuate CS-induced lung inflammation and injury by inhibiting aberrantly activated AMs pyroptosis. In this present study, the profiles of AMs pyroptosis, the effects of ADSCs-Exo on AMs pyroptosis in addition to the roles of ADSCs-Exo in ameliorating lung inflammation and injury in CS-induced lung inflammation and injury were investigated in CS-stressed mice and in CS extract (CSE)-stressed AMs with properties modelling those found in CS-induced lung inflammation and injury and COPD.

## Methods

All protocols and procedures were approved by the Research Ethic Committee of the Second Affiliated Hospital of Fujian Medical University (2021-351). All animal handling procedures were approved by the Animal Care and Use Committee, Quanzhou Medical College (SYXK 2016-0001).

### Primary hADSCs cultivation and identification

Primary ADSCs were isolated from the abdominal subcutaneous adipose tissues harvested from three healthy donors (Table 1) and cultured in DMEM media containing 10% Fetal Bovine Serum (FBS, Invitrogen, NY, USA) as described in previous studies [17]. Cells were passaged when reaching 80% of confluence and used at passages three to six. The immunophenotypes of cells were analyzed via detecting the profiles of stem cell surface markers, including CD44 and CD90, and that of hematopoietic markers, including CD34 and CD45 (5 μL/10^6^ cells; Biolegend, California, USA), through a NovoCyte flow cytometer (Agilent, California, USA). In addition, cells were cultured with adipogenic, osteogenic and chondrogenic differentiation media, then the cellular differentiation potential was determined by Oil red O staining, Alizarin Red Staining and Alcian blue staining, respectively (STEMCELL Technologies, Vancouver, Canada).

**Table 1:** The demographic information of adipose tissues donors

Number	Age	Gender	Weight (kg)	Height (cm)	BMI (kg/m^2^)
1	26	Female	59	169	20.7
2	24	Female	56	163	21.2
3	27	Female	51	159	20.2

### Exosomes isolation

When ADSCs reached 70% of confluence, the media was discarded and replaced by fresh media with Exo-depleted FBS. Then, ADSCs were incubated for another 24 h and the media were collected subsequently. Exo were isolated from these media at 4 °C by an Exo-isolation kit (Invitrogen, California, USA) according to the manufacturer’s instructions. Briefly, 20 mL culture media were successively spun at 3000*g* for 10 min and at 10,000*g* for 20 min using a high-speed centrifuge (3K15, Sigma Laborzentrifugen, Freistaat Sachsen, Germany). Then the supernatant was mixed with 10 mL Exo concentration solution followed by being vortexed and incubated for 2 h. Exo pellets were precipitated by centrifuging the mixture at 10,000*g* for 60 min. The pellets were resuspended with PBS and purified with Exo purification filters at 3000*g* for 10 min. Finally, the pellets were resuspended in 200 μL sterile PBS and stored at − 80 °C. Exosomal protein concentration was quantified by a BCA kit (MilliporeSigma, Burlington, USA) and adjusted to 100 mg/L using sterile PBS prior to use.

### Exosomes identification

Freshly isolated ADSCs-Exo in PBS were adsorbed at carbon-coated copper grids (MilliporeSigma, Burlington, USA) for 5 min and the excess was blotted on filter paper (Thermo Fisher Scientific, Waltham, USA). Subsequently, these Exo were stained with 3% uranyl acetate for 5 min and the excess was blotted. The size and morphology of Exo were examined by a transmission electron microscope (Hitachi, Tokyo, Japan). In addition, the size distribution of these Exo was determined by a high-sensitivity nano-flow cytometer (NanoFCM, Fujian, China) as described previously [30]. Moreover, the profiles of exosomal markers, including Flotillin, CD9 and CD81, and that of Calnexin (1:1000; CST, Boston, USA), an endoplasmic reticulum marker, were detected by western blot. The expressions of CD9 and CD81 (at 1:2 dilution using staining buffer) were further analyzed by flow cytometry.

### In vivo study design

Twenty-four age-matched (8-week-old) male C57BL/6 mice, one frequently used inbred mouse strain, were purchased from Huazhong Agricultural University (SCXY, 2015-0019) and kept under specific pathogen-free conditions. They were randomly assigned in equal numbers to three groups, which are the control group, CS-stressed group and CS-ADSCs-Exo group, respectively. In vivo model was established in mice by exposing to CS for 4 weeks as previously described [31–33]. CS was generated by the combustion of commercial cigarettes (11 mg tar, 1.2 mg nicotine and 13 mg carbon monoxide; Hongtashan, Yunnan, China). Mice were exposed to room air or to CS (10 cigarettes/exposure, twice a day with 30-min smoke-free intervals) in whole-body exposure chambers for 4 weeks. On the next day, mice in the CS-ADSCs-Exo group were intratracheally treated with 30 μL ADSCs-Exo while those in the remaining two groups were treated with 30 μL sterile PBS. Animals were monitored daily and their body weights were recorded weekly. 4 weeks after the treatment of ADSCs-Exo or PBS, animals were euthanatized and samples were harvested.

### CSE preparation

CSE was made from cigarette smoke following a published protocol [34] with minor modifications. Briefly, three filter-free aforementioned cigarettes were burned and the smoke was slowly bubbled into a 15-mL tube (Corning, NY, USA) containing 5 mL sterile and serum-free RPMI-1640 media (HyClone, Logan, USA). This media was regarded as the 100% CSE solution. Subsequently, this 100% CSE solution was adjusted to pH 7.4 followed by being filtered by 0.22-µm filters (MilliporeSigma, Burlington, USA) to remove insoluble particles and microorganisms and being diluted in the culture media containing 10% FBS prior to use.

### Ex vivo study design

After euthanasia, tracheotomy was performed followed by tracheal tube insertion and BALF collection. Subsequently, these BALF samples were centrifuged at 300*g* at 4 °C for 10 min to pellet the cellular contents of BALF. The cell pellet was resuspended in 1 mL haemolysis buffer for 2 min at room temperature to lyse residual erythrocytes. The centrifugation step was repeated and the remaining cell pellet was resuspended in 5 mL RPMI-1640 media supplemented with 10% FBS and then was seeded in a culture flask. To allow AMs to adhere to the flask, these cells were incubated in a 37 °C incubator for 3 h. Then the media was discarded and the cells were washed with PBS to remove non-adherent cells. The adherent cells were mouse alveolar macrophages (MAMs). Notably, MAMs harvested from mice in the control group, CS-stressed group and CS-ADSCs-Exo group are treated as the control MAMs, CS-stressed MAMs and CS-ADSCs-Exo MAMs, respectively.

### In vitro study design

A CSE-induced model was used for the in vitro section. MH-S cells, one commonly used continuous MAMs cell line, was purchased from ATCC (CRL-2019™, Manassas, USA) and cultured in RPMI-1640 media with different concentrations of CSE for 24 h followed by measuring the cellular viability via CCK-8 assay (Abcam, Cambridge, UK), and 2% was determined as the optimum experimental concentration (data not shown). MH-S cells were randomly divided into three groups, which were the control group, the CSE-stressed group and the CSE-ADSCs-Exo group, respectively. As for the control group, MH-S cells were cultured in normal media for 24 h, the media were discarded and cells were cultured in fresh normal media for another 24 h. As for the CSE-stressed group, MH-S cells were cultured in fresh normal media for 24 h following the stimulation of 2% CSE for 24 h. As for the CSE-ADSCs-Exo group, MH-S cells were induced by 2% CSE for 24 h and then cultured in fresh media containing 50 μL ADSCs-Exo (5 μg of total exosomal protein) for another 24 h. Cells and culture media were collected.

### Pro-inflammatory cell counts analysis

The total pro-inflammatory cell population was determined via a hemocytometer as previously described [35] and the differential cells were analyzed on 300 cells stained with Diff-Quik (Canadawide Scientific, Ontario, Canada).

### Pyroptosis assay

The pyroptosis levels of macrophages were determined by detecting the expression profiles of pyroptosis-indicated proteins, including Caspase-1 (1:1000), ASC (1:1000), GSDMD (1:1000) and NLRP3 (1:50; Abcam, Cambridge, UK) through western blot. GAPDH (1:500; Biolegend, California, USA) was used as loading control.

### Phagocytosis assay

The phagocytic activity of AMs was determined by flow cytometry according to previous research [36]. MAMs and MH-S in all groups were seeded in 6-well plates at 1 × 10^6^ cells/well and cultured for 24 h. Subsequently, these cells were incubated with fluorescent bio-particles (Polysciences, Warrington, USA) at a cell to particle ratio of 1:10 at 37 °C for 2 h. Thereafter, these cells were washed by sterile PBS three times followed by being digested by trypsin and resuspended in 500 μL PBS. A NovoCyte flow cytometer was set up with precise parameters, including pulse height, to allow macrophages to be distinguished from other cells. Thereafter, the fluorescence intensity was detected.

### ELISA assay

The profiles of pro-inflammatory mediators, including TNF-α, IL-6 and CXCL1, in BALF were determined by ELISA assay according to the manufacturer’s instructions (R&D Systems, Minnesota, USA). Absorbance was measured by a spectrophotometer (Thermo Fisher Scientific, Waltham, USA).

### Western blot

The total proteins were extracted by lysing macrophages (MAMs and MH-S) and ADSCs-Exo in RIPA lysis buffer supplemented with protease and phosphatase inhibitors (Thermo Fisher Scientific, Waltham, USA). Subsequently, the protein lysates concentrations were determined by BCA assay. Equal amounts of total proteins were separated by sodium dodecyl sulphate-polyacrylamide gel electrophoresis and then electrophoretically transferred to polyvinylidene difluoride membranes (Bio-Rad, Hercules, USA). These membranes were blocked with 5% skimmed milk for 1 h at room temperature, and then were incubated with rabbit anti-mouse Caspase-1, ASC, GSDMD, NLRP3 (Abcam, Cambridge, UK) and GAPDH (Biolegend, California, USA) primary antibodies overnight at 4 °C. Afterwards, the membranes were washed and incubated with goat anti-rabbit horseradish peroxidase-conjugated secondary antibody (Beyotime, Shanghai, China) for 1 hour at room temperature. Target proteins were visualized via an enhanced chemiluminescence detection reagent (Bio-Rad, Hercules, USA) and were determined by an Image Quant LAS4000 chemiluminescence detection system (GE Healthcare Systems, Chicago, USA). Images were analyzed using the Quantity One software (Bio-Rad, Hercules, USA). GAPDH was used as normalization control.

### Flow cytometry

Suspensions of hADSCs or Exo were prepared and incubated with FITC-or PE-conjugated antibodies or their corresponding isotype control antibodies at 4 °C for 30 min. Subsequently, these samples were processed by a NovoCyte flow cytometer (Agilent, California, USA) and images were analyzed by the FlowJo software.

### Immunofluorescence staining

The left lung was fixed with formalin and embedded in paraffin. Subsequently, 4-μm-thick sections were prepared. AMs were seeded on cohesive coverslips followed by fixing with 4% paraformaldehyde for 10 min and permeabilizing with 0.1% Triton X-100. After antigen retrieval by heating the samples in antigen retrieval buffer, lung sections or fixed cells were blocked in goat serum for 1 h. Then these samples were incubated with primary antibodies overnight at 4 °C. After washing, sections or cells were stained with fluorophore-conjugated second antibodies for 1 h at 37 °C. Then these samples were washed again and observed by a confocal laser scanning microscope (Olympus, Tokyo, Japan).

### Histology analysis

Lung sections were processed for standard hematoxylin and eosin staining. Images were taken under an optical microscope (Olympus, Tokyo, Japan) and blindly analyzed by two experienced pathologists independently.

### Mucus secretion assessment

Lung sections were processed for standard Alcian blue (AB)-periodic acid Schiff (PAS) staining. A blue stain indicates the presence of mucus proteins. The secretion of mucus was quantified by Image-Pro Plus 6.0 (Media Cybernetics, Rockville, USA) and the results were expressed as fold increase over control.

### Statistical analysis

Statistical analysis was performed using SPSS22.0 (IBM Analytics, NY, USA). Data were represented as mean ± standard deviation and analyzed by independent-samples t-test and one-way ANOVA, as applicable. *P* values < 0.05 were considered as statistical significance.

## Results

### Characterization of ADSCs

**Fig. 1:**
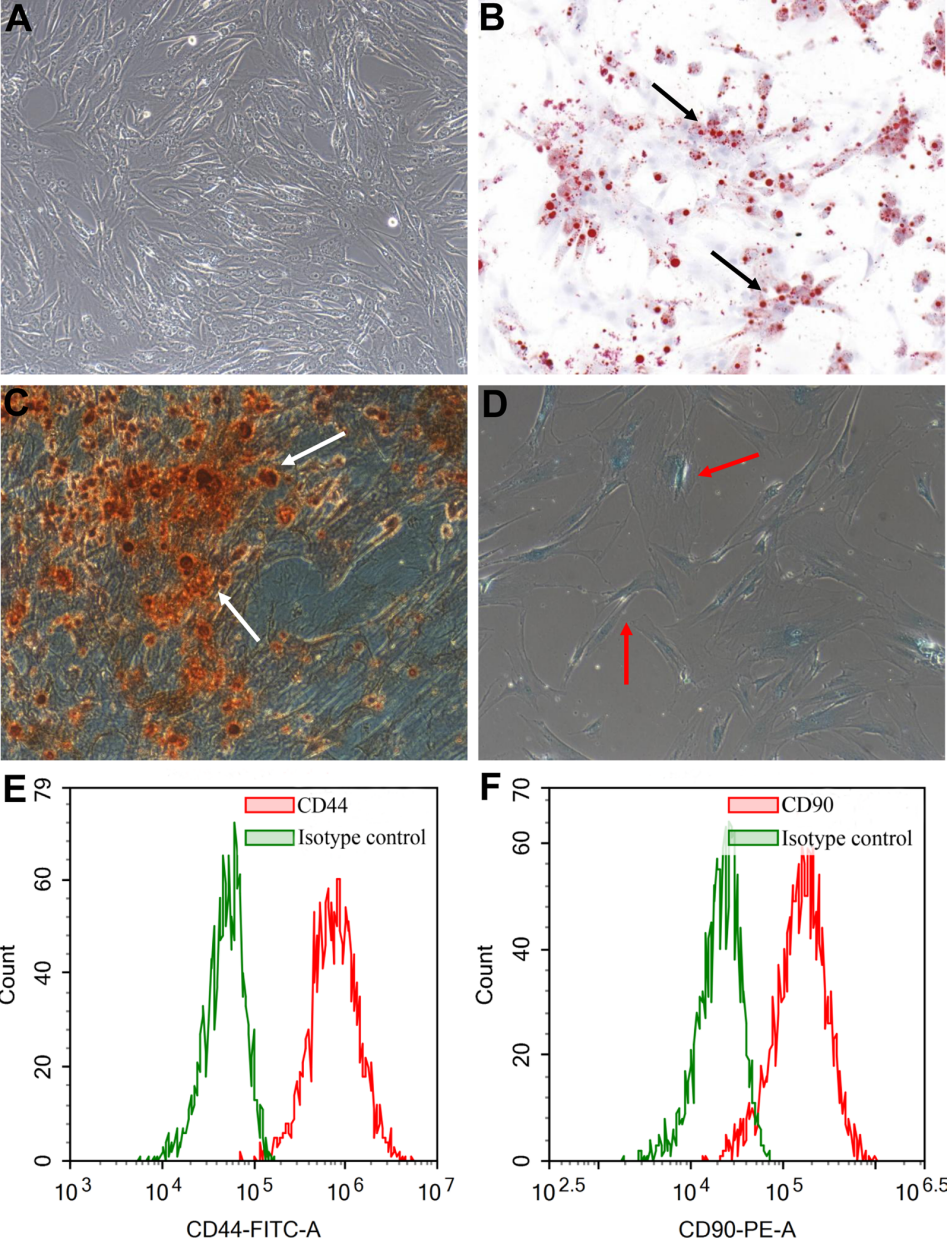
Primary culture and multiple differentiation of ADSCs. These spindle-shaped cells isolated from human adipose tissue adhered to the Petri dishes and formed a monolayer (**A**). ADSCs differentiated into adipocytes as lipids were accumulated in ADSCs culture. Black arrows indicate the presence of lipids droplets (**B**). ADSCs differentiated into osteoblasts as extracellular calcium phosphate deposits were observed in ADSCs culture. White arrows indicate the presence of osteogenic mineral deposition (**C**). ADSCs differentiated into chondroblasts as nodules were formed in ADSCs culture. Red arrows indicate the presence of nodules and inter chondrogenic nodule connections (**D**). ADSCs were positive for CD44 (**E**). ADSCs were positive for CD90 (**F**)

The cells isolated from human subcutaneous adipose tissues showed a spindle-shape and adhered to the petri dishes, forming a monolayer (Fig. 1A). The Oil Red O staining, Alizarin Red Staining and Alcian Blue staining showed that there were many lipid droplets in the cytoplasm and mineralized nodules and glycosaminoglycans in the extracellular matrix, indicating that these cells were able to differentiate into adipocytes (Fig. 1B), osteocytes (Fig. 1C) and chondrocytes (Fig. 1D) after the induction of adipogenic, osteogenic, and chondrogenic differentiation media. In addition, flow cytometry demonstrated that these cells showed standard MSCs phenotypes: highly positive for stem cell surface markers, CD44 (90.03%, Fig. 1E) and CD90 (99.29%, Fig. 1F) and negative for hematopoietic markers, CD34 (0.56%, Additional file 1: Fig. S1A) and CD45 (0.07%, Additional file 1: Fig. S1B). The demographic information of the donors of the adipose tissues used in the study was presented in Table 1. These results indicated that these cells are matching with the criteria of MSCs [18] and should be regarded as ADSCs.

### Identification of ADSCs-Exo

**Fig. 2:**
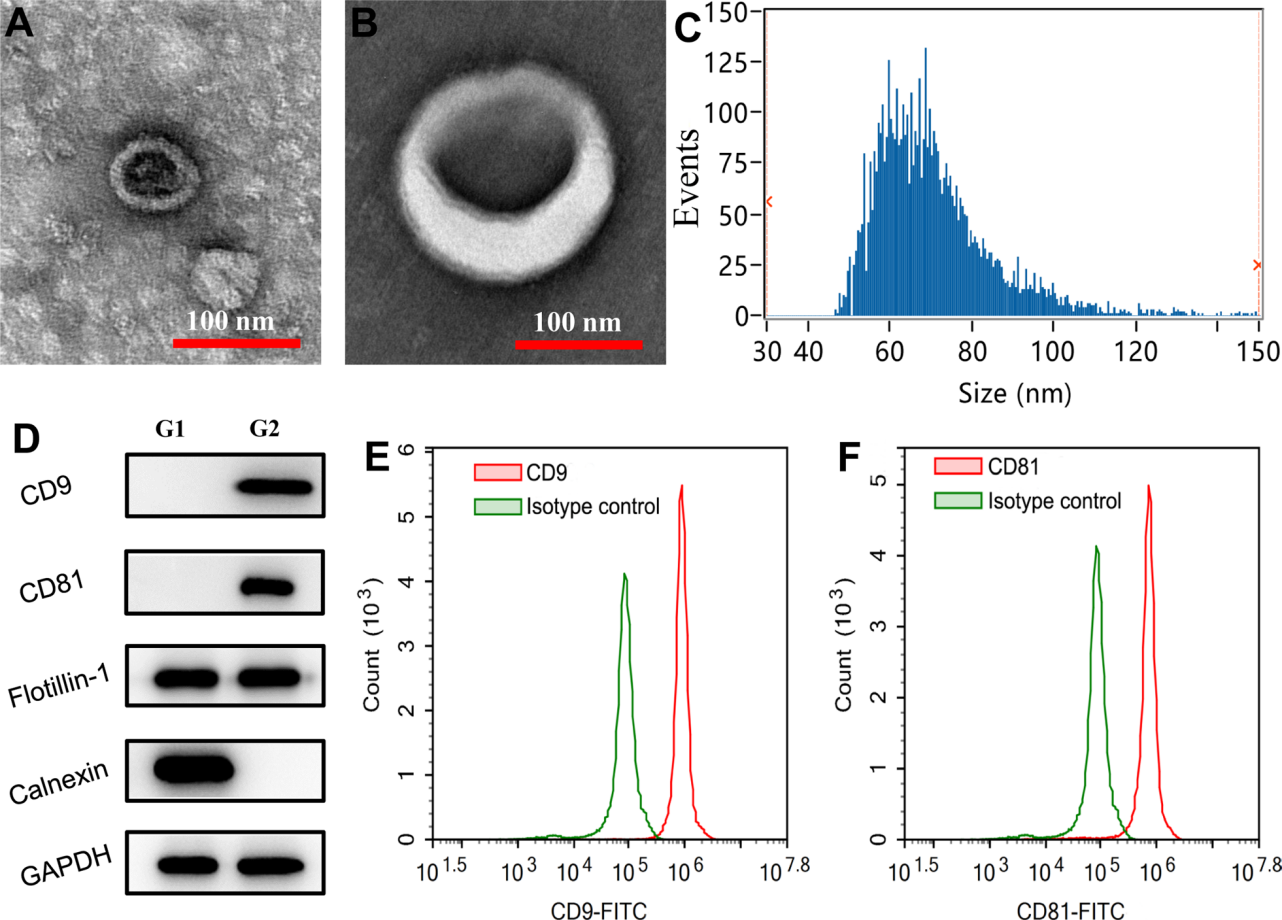
Characterization of ADSCs-Exo. The ADSCs-Exo observed by TEM showed a typical cup-shape (**A**, **B**). The representative nano-flow cytometer histogram of ADSCs-Exo (**C**). Western blot indicated that ADSCs-Exo were positive for CD9, CD81 and Flotillin, but they were negative for Calnexin (**D**). Flow cytometry showed that most of the ADSCs-Exo were positive for CD9 (**E**) and CD81 (**F**). G1 refers to ADSCs and G2 refers to ADSCs-Exo

The pellets isolated from the culture media of hADSCs showed typical cup-shape on TEM images (Fig. 2A, B). The representative nano flow cytometry histogram showed that the sizes of these pellets were within the reported range of Exo, with a mean of 70.67 nm (Fig. 2C). In addition, western blot demonstrated that these pellets were positive for exosomal markers, including CD9, CD81 and Flotillin but were negative for Calnexin protein (Fig. 2D). Flow cytometry showed that CD9 (96.05%) and CD81 (95.19%) were expressed in most of these pellets (Fig. 2E, F). These findings indicated that these pellets are ADSCs-Exo, and these Exo are highly purified.

### Treatment with ADSCs-Exo facilitated body weight gain in CS-stressed mice 

**Fig. 3:**
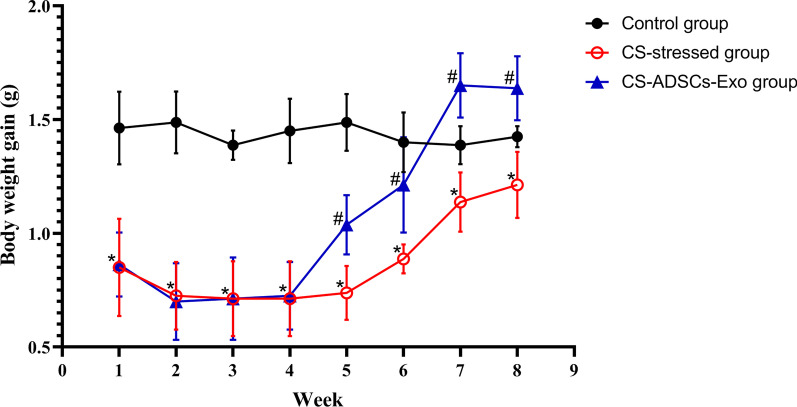
Effects of ADSCs-Exo on body weight gain in mice exposed to CS for 4 weeks. Values are represented as the mean ± standard deviation (n = 8). *P < 0.05 vs. control; ^#^*P* < 0.05 vs. ADSCs-Exo-treated

To investigate the effects of ADSCs-Exo on the improvement of the overall conditions of mice, animals’ body weights were monitored. The mean starting body weight of mice in three groups was 18.40 g, 18.46 g and 18.39 g (*P* > 0.05), respectively. As shown in Fig. 3, although mice’ body weights were similar before being exposed to air or CS, mice in the CS-stressed group and CS-ADSCs-Exo group gained significantly less weights than those in the control group after a 1-week exposure period (*P* < 0.05). The treatment of ADSCs-Exo remarkably enhanced the weight gain of CS-stressed mice compared with CS-stressed mice received PBS treatment (*P* < 0.05).

### Treatment with ADSCs-Exo relieved CS-induced lung inflammation

**Fig. 4:**
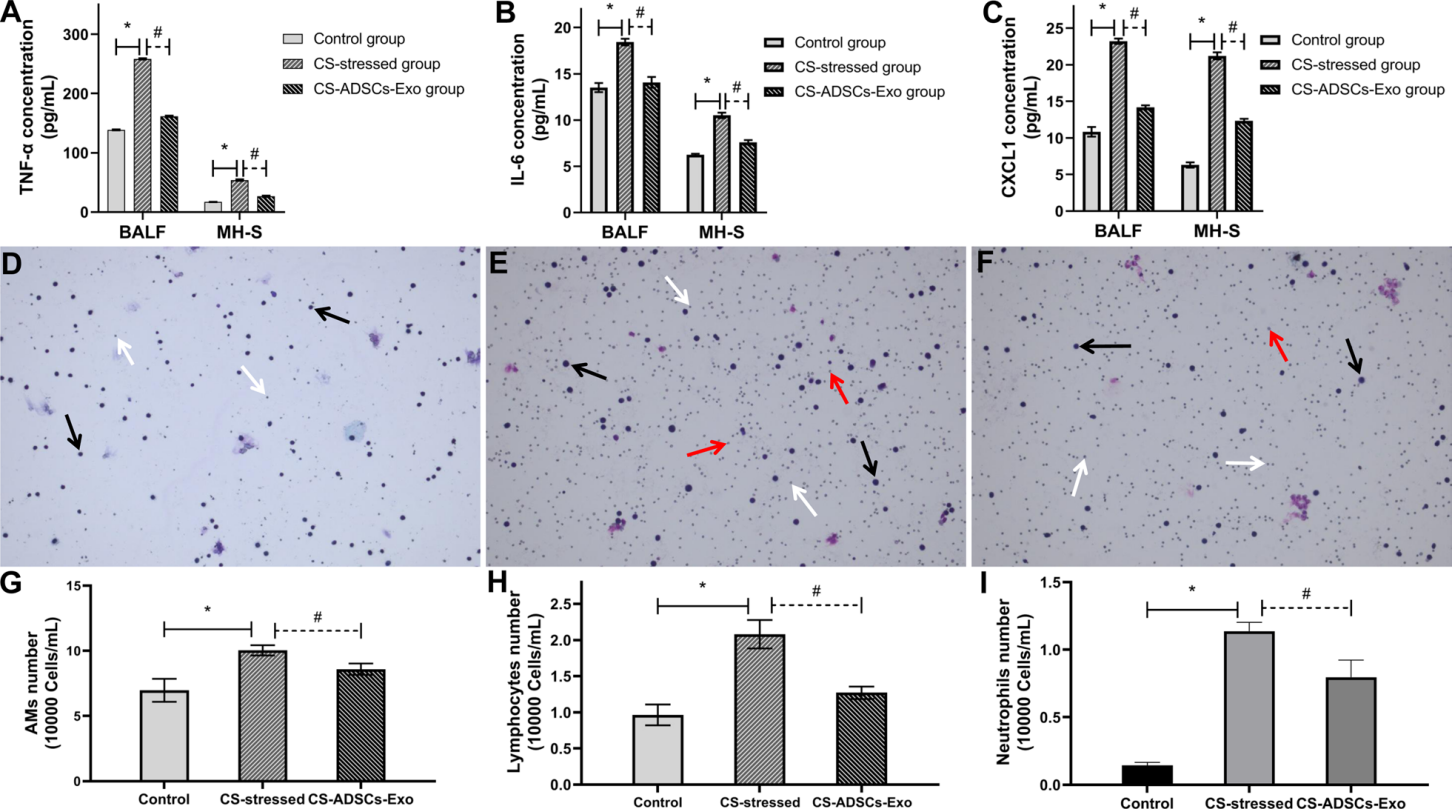
ADSCs-Exo mitigated CS-induced pulmonary inflammation. The levels of pro-inflammatory mediators in the BALF and the supernatant of MH-S cells, including TNF-α (**A**), IL-6 (**B**) and CXCL1 (**C**), were determined by ELISA. Diff-Quik staining was performed to analyse the populations of AMs (black arrows), neutrophils (red arrows) and lymphocytes (white arrows) in the BALF (**D**–**G**). *P < 0.05 vs. control; ^#^*P* < 0.05 vs. CS-ADSCs-Exo

To evaluate whether ADSCs-Exo is capable of reducing the profile of inflammation in the lungs in response to CS exposure, the levels of pro-inflammatory mediators in BALF and MH-S cell supernatant and the populations of inflammatory cells in BALF were determined. As depicted in Fig. 4A–C, the levels of TNF-α, IL-6 and CXCL1 were significantly increased in the CS-stressed group compared with the control group (*P* < 0.05). Conversely, the CS-induced elevations of TNF-α, IL-6 and CXCL1 in BALF were markedly reduced by the administration of ADSCs-Exo (*P* < 0.05). After Diff-Quik staining, those large cells (around 20 μm) with pseudopods, violet nuclei, light blue cytoplasm and light purple granules were treated as AMs, those small cells (6–8 μm) with dark purple nuclei and little to no cytoplasm (light purple) were treated as lymphocytes and those cells (12–15 μm) with blue nuclei (with typical lobed appearance), pink cytoplasm and violet granules were treated as neutrophils. As demonstrated in Fig. 4D–I, the populations of BALF pro-inflammatory cells, including AMs (*P* < 0.001), lymphocytes (*P* < 0.01) and neutrophils (*P* < 0.05) were significantly increased in CS-stressed mice compared with the control mice. The populations of AMs, lymphocytes and neutrophils in CS-stressed mice greatly declined following ADSCs-Exo treatment (*P* < 0.05).

### Treatment with ADSCs-Exo attenuated CS-induced lung injury and mucus hypersecretion

**Fig. 5:**
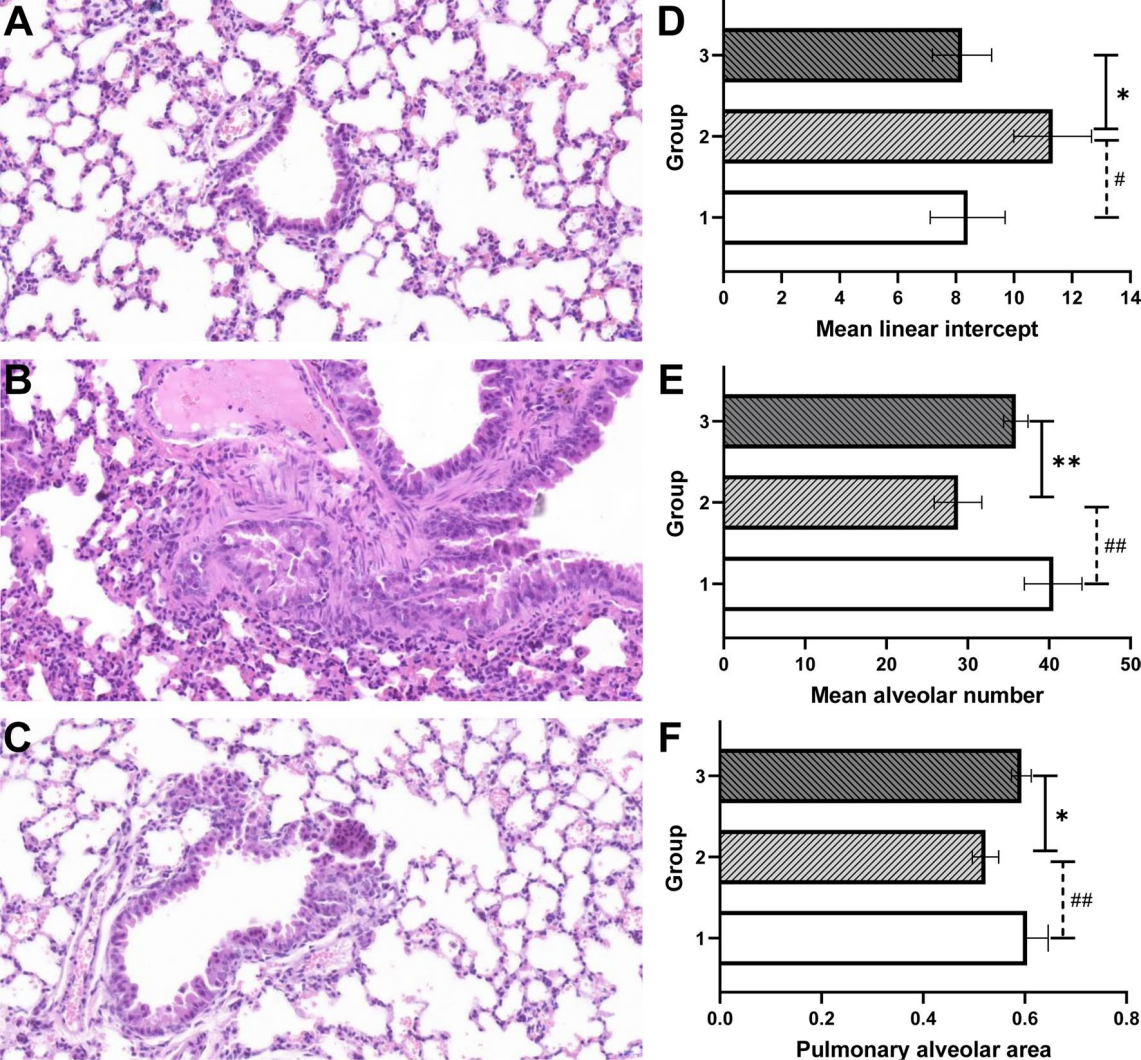
ADSCs-Exo attenuated CS-induced lung injury. Lung tissues of mice from each group were stained with hematoxylin and eosin (n = 4). Images of stained lung sections of control (**A**), CS-stressed (**B**) and CS-ADSCs-Exo (**C**) groups. The quantitative analysis results of pulmonary emphysema indicators, including the mean linear intercept (**D**), mean alveolar number (**E**) and pulmonary alveolar area (**F**). Group 1, group1 and group 3 refer to the control group, CS-stressed group and CS-ADSCs-Exo group, respectively.*P < 0.05 vs. control; **P < 0.01 vs. control; ^#^*P* < 0.05 vs. CS-ADSCs-Exo; ^##^*P* < 0.01 vs. CS-ADSCs-Exo

**Fig. 6:**
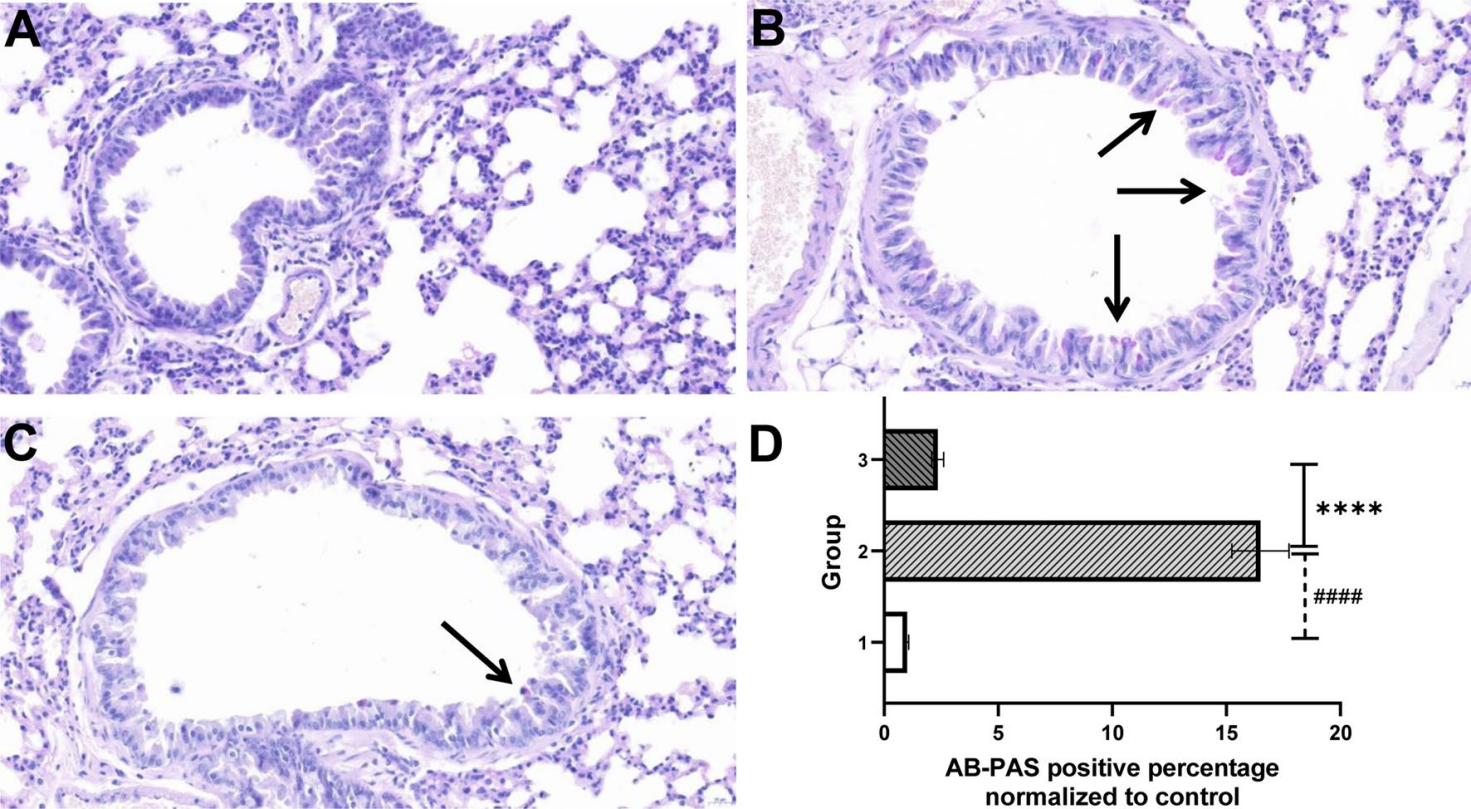
ADSCs-Exo attenuated CS-induced mucus hypersecretion. Lung tissues of mice from each group were stained with AB-PAS (n = 5). Images of stained lung sections of control (**A**), CS-stressed (**B**) and CS-ADSCs-Exo (**C**) groups. The quantitative analysis result of mice mucus production in each group (**D**). Arrows in **B** and **C** indicate the presence of mucus proteins. Group 1, group1 and group 3 refer to the control group, CS-stressed group and CS-ADSCs-Exo group, respectively. ****P < 0.001 vs. control; ^####^*P* < 0.001 vs. CS-ADSCs-Exo

To study whether ADSCs-Exo is able to repair CS-induced pulmonary destruction, lung architecture was observed by hematoxylin and eosin staining. As shown in Fig. 5A–C, lung structure in the control group was normal without inflammatory cell infiltration. In contrast, CS exposure gave rise to excessive inflammatory cell infiltration and obvious architecture destruction in lungs, including alveoli enlargement and fracture, bronchial mucosal and vessel walls thickening, epithelial cilia reduction and epithelial cells hyperplasia. In addition, the exposure of CS increased the mean linear intercept while reduce the mean alveolar number and the pulmonary alveolar area of lung, indicating the presence of pulmonary emphysema (Fig. 5D–F). These lung alterations induced by CS exposure were remarkably alleviated by the treatment of ADSCs-Exo. To assess the effects of ADSCs-Exo on CS-induced airway mucus production, mucous glycoconjugates were visualized by the AB-PAS staining and were quantified subsequently. As shown in Fig. 6A–D, CS exposure significantly increased airway mucus secretion, however, the treatment of ADSCs-Exo effectively reduced the production of mucus.

### Treatment with ADSCs-Exo reversed the phagocytic activity of CS/CSE-stressed AMs

**Fig. 7:**
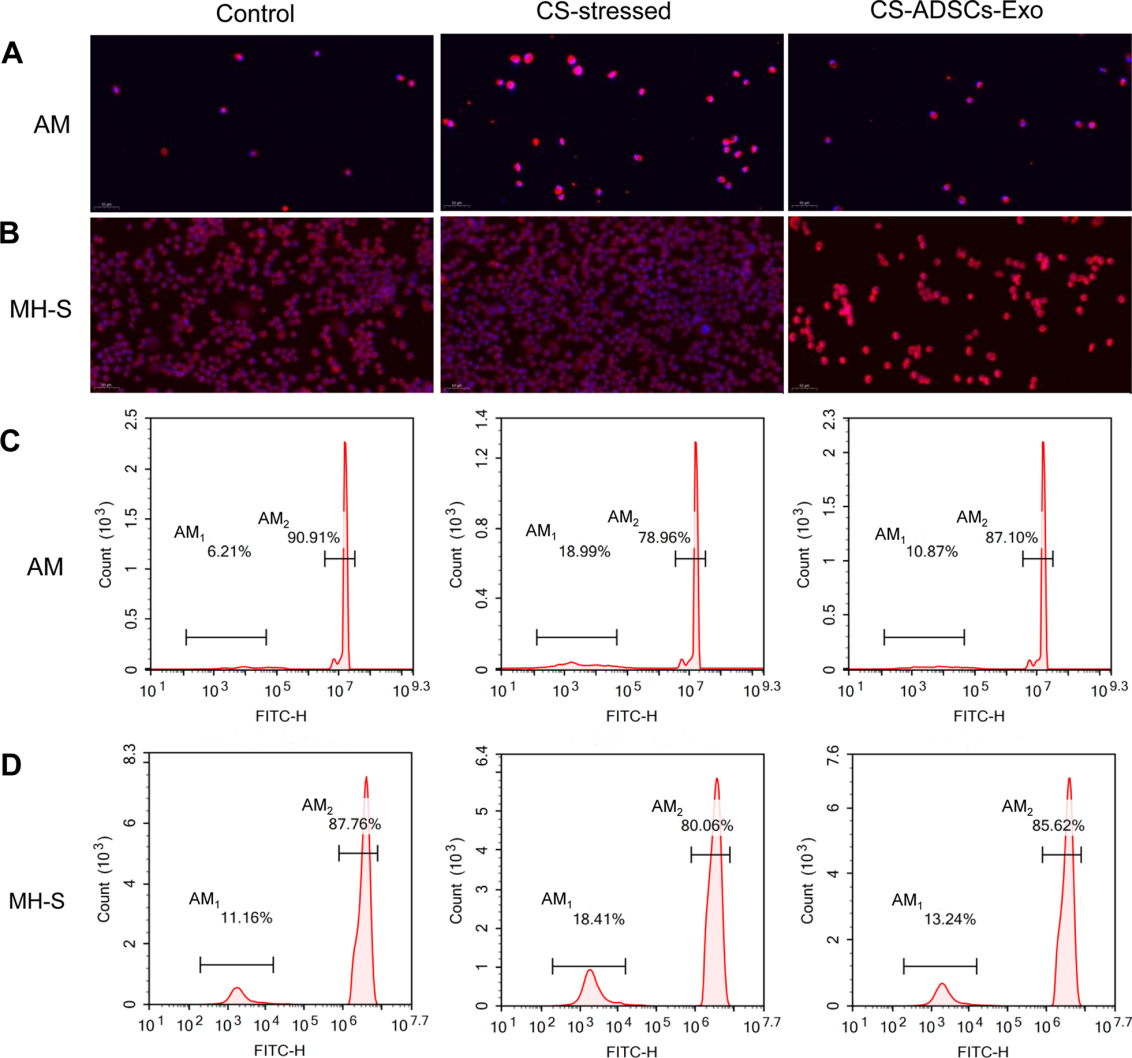
ADSCs-Exo reversed the phagocytic activity of AMs. The AM cell marker F4/80 was visualized by immunofluorescence (**A**, **B**), and flow cytometry was used to measure AM phagocytic activity (**C**, **D**). Phagocytic activity assay was repeated for 3 times

The immunofluorescence staining indicated that both the isolated MAMs and the commercial AM cell line, namely MH-S, showed classical macrophage shapes and are positive for F4/80, which are the hallmarks of AMs, indicating that these cells are typical AMs (Fig. 7A, B). Flow cytometry was applied to investigate the roles of ADSCs-Exo in the phagocytic ability of AMs following CS exposure. As shown in Fig. 7C, D, compared to the control group, the proportion of BALF AMs that without phagocytic activity (AM_1_) greatly increased while the percentage of BALF AMs that possess phagocytic activity (AM_2_) remarkably decreased in CS-stressed group (*P* < 0.001). Excitingly, ADSCs-Exo treatment effectively reduced the AM_1_ to AM_2_ ratio (*P* < 0.01). Similarly, in vitro, the stimulation of CSE led to an obvious alteration of MH-S cells from the AM_1_ phenotype to the AM_2_ phenotype (*P* < 0.001). However, the impaired phagocytic activity of MH-S cells was markedly restored following the administration of ADSCs-Exo (*P* < 0.001).

### Treatment with ADSCs-Exo inhibited the pyroptosis level of CS/CSE-stressed AMs

**Fig. 8:**
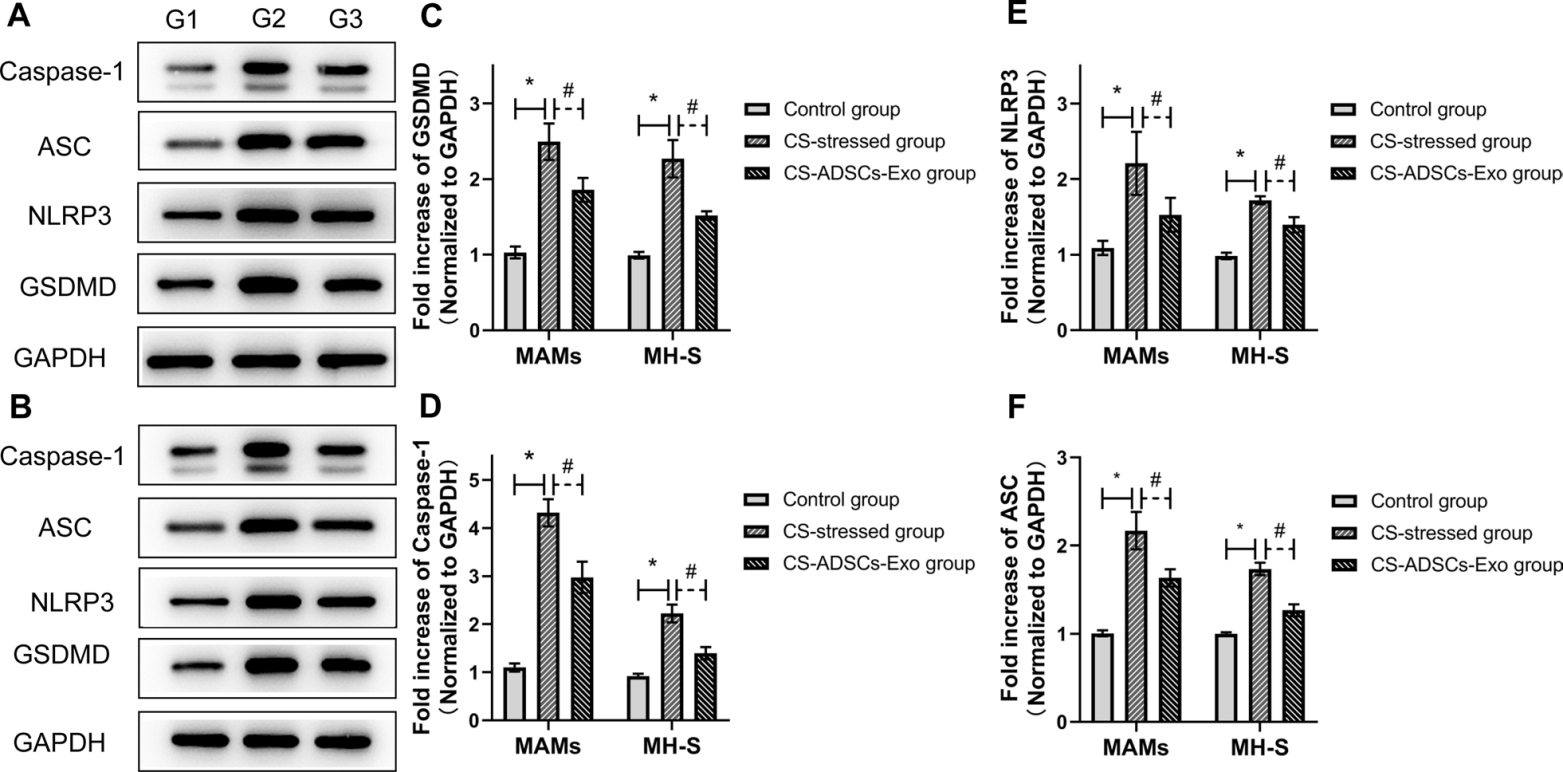
ADSCs-Exo inhibited the level of pyroptosis in AMs. The expression levels of pyroptosis-indicated proteins were analyzed by western blot in AMs isolated from the BALF (**A**) and in MH-S cells (**B**). The group mean data of GSDMD (**C**), Caspase-1 (**D**), NLRP3 (**E**) and ASC (**F**) in BALF AMs ( n = 6) and MH-S cells ( n = 3). Group 1, group1 and group 3 refer to the control group, CS-stressed group and CS-ADSCs-Exo group, respectively. *P < 0.05 vs. control; ^#^*P* < 0.05 vs. CS-ADSCs-Exo

To demonstrate the effects of ADSCs-Exo on pyroptosis of CS/CSE-stressed AMs, western blot was applied to determine the profiles of pyroptosis-indicated proteins. As shown in Fig. 8A and C–F, in vivo, CS exposure increased the expressions of NLRP3, caspase-1, ASC and GSDMD in AMs while their expressions were significantly reduced by the treatment of ADSCs-Exo (*P* < 0.05). Consistently, as illustrated in Fig. 8B and C–F, in vitro, the levels of these pyroptosis markers greatly increased in MH-S cells in response to CSE stimulation, while the elevation induced by CSE was significantly reduced by the treatment of ADSCs-Exo (*P* < 0.05).

## Discussion

COPD has been posing a heavy burden worldwide [37], however, precise and efficient therapeutic strategies that can halt or reverse disease progression are still not available or scarce [5, 38]. MSCs have shown promise in tissue repair and regeneration after injury [8]. The extracellular vesicles derived from MSCs harbors therapeutic potential comparable to the cells of origin, moreover, the treatment of MSCs-derived extracellular vesicles is superior to that of MSCs in many aspects, such as targeted delivery, low immunogenicity, high repairability [39] and treatment safety and fewer ethical concerns, therefore, there is an increasing interest in investigating the protective roles of MSCs-derived extracellular vesicles, especially Exo, in many diseases, including COPD [22, 40, 41]. Although ADSCs-Exo have initially shown therapeutic promise in elastase-induced COPD [22], very little is known about their roles in lung inflammation and injury caused by CS and the underlying mechanisms.

Considering that CS is the predominant cause of COPD [1], a CS-stressed mice model (in vivo) and a CS-stressed MH-S model (in vitro) were used in this research. Considering that the C57BL/6 mouse is a susceptible strain in developing CS-induced COPD, C57BL/6 mice were used for this study [42]. On one hand, we demonstrated that CS exposure and CSE stimulation significantly elevated the level of pyroptosis in AMs, however, they obviously inhibited the phagocytosis of AMs, resulting in persistent inflammation, mucus overproduction and structural disruption in lung tissues. On the other hand, we also found that the treatment of ADSCs-Exo substantially attenuated CS-induced AMs pyroptosis, thereby reducing inflammatory cell infiltration, inhibiting the inflammatory response and rescuing tissue architectural destruction in the lungs in CS-stressed mice. Our findings point the way towards a potential treatment target and a promising treatment option for CS-induced lung inflammation and injury.

Firstly, this study was conducted to decipher the potential therapeutic roles of ADSCs-Exo in CS-induced lung inflammation and injury. Nutritional depletion is a major feature of COPD and reduced body weight gain has been widely investigated [43]. Notably, undernutrition is an adverse factor of COPD and it is associated with declining survival time. Moreover, it is also a debilitating feature of COPD, which indicates a high risk of acute exacerbation [44]. This present study showed that mice became poorly nourished in response to CS exposure. Loss of appetite and growth retardation were observed in CS-stressed mice. Excitingly, the treatment of ADSCs-Exo greatly enhanced the appetite, food intake and body weight gain in mice with CS exposure, implying that ADSCs-Exo may hold promise for improving the nutritional status and overall condition of CS-stressed mice. Persistently elevated airway inflammation levels induced by infiltrating pro-inflammatory cells, including AMs, lymphocytes and neutrophils, greatly contribute to the development of COPD [3, 45]. Pro-inflammatory mediators and destructive enzymes released by these immune cells are largely involved in progressive destruction and dysregulated remodelling of the lung structure in COPD [46, 47]. In this research, we found that the populations of AMs, lymphocytes and neutrophils in the BALF were significantly increased after CS exposure, however, these increases were remarkably reduced following ADSCs-Exo treatment. TNF-α, IL-6 and CXCL1 are vital pro-inflammatory mediators, which are upregulated in COPD, and they greatly amplify the pulmonary inflammatory response [46]. This research demonstrated that the elevated levels of TNF-α, IL-6 and CXCL1 induced by CS exposure were markedly decreased following the treatment of ADSCs-Exo. In agreement with the structural abnormalities caused by relatively short duration of CS exposure described previously [33], we found that the pulmonary architecture was obviously disrupted by 4 weeks of CS exposure. Additionally, pulmonary emphysema caused by CS exposure was also observed. Notably, treatment with ADSCs-Exo greatly mitigated lung injury caused by CS. Chronic pulmonary inflammation also leads to goblet cell hyperplasia, resulting in airway mucus overproduction and hypersecretion [48], thereby greatly increasing the mortality risk of COPD [33, 48]. This present research for the first time revealed the ability of ADSCs-Exo in reducing the production of intracellular mucus glycoconjugates induced by CS. These aforementioned findings indicated that ADSCs-Exo were able to ameliorate CS-induced pulmonary inflammation, mitigate lung injury and reduce mucus secretion.

Then the question arose about how ADSCs-Exo exert their protective roles in CS-induced lung inflammation and injury. AMs are the most abundant pro-inflammatory cells in the lungs, and they play a pivotal role in maintaining pulmonary homeostasis [49]. Moreover, in COPD, AMs also represent key orchestrators of chronic airway inflammation [46]. Even though the number of AMs was increased in COPD, their functions were severely impaired [25]. Notably, AMs phagocytosis, which is central to the attenuation and resolution of pulmonary inflammation, was obviously inhibited in COPD [50]. Similar to previous studies, our present study showed that CS exposure and CSE stimulation greatly decreased the phagocytic activity of AMs. However, the defective phagocytic activity of AMs was significantly restored by the treatment of ADSCs-Exo. Apart from functional alterations, a variety of cell death pathways were also activated in AMs in COPD [51]. Pyroptosis, a pro-inflammatory programmed cell death pathway driven by several inflammatory caspases (caspase-1 and caspase-11/4/5), is widely involved in many inflammatory airway diseases, including COPD [28]. In COPD, the level of caspase-1-dependent pyroptosis was elevated and the inhibition of canonical pyroptosis effectively protected mice against CS-induced pulmonary inflammation response and injury [52]. The initiation of canonical pyroptosis relies on the activation of the NLRP3 inflammasome comprising by NLRP3, ASC, and activated caspase-1 [28]. Activation of the inflammasome involves the priming of gasdermins, including gasdermin D (GSDMD), the major executor of pyroptosis, and the cleavage of pro-inflammatory mediators [53]. A previous study conducted by Di Stefano and colleagues has shown that the NLRP3 inflammasome was not activated in stable COPD [54], thus, pyroptosis may act more on the initial stages and acute exacerbations of COPD. Consistent with previous investigations, our study showed that the levels of these pyroptosis-associated proteins were significantly upregulated in CS/CSE-stressed AMs. Importantly, both in vivo and in vitro, these elevations were markedly attenuated by the treatment of ADSCs-Exo, implying the ability of ADSCs-Exo in offering protection against AMs pyroptosis caused by CS or CSE.

However, there are some limitations to this study. In this study, we did not explore the ventilation parameters in mice and CS-stressed mice only received 4 weeks of CS exposure, which only represents the early stage of COPD [55]. In addition, the severity of CS-induced COPD is only equivalent to that of early-stage COPD in humans based on the Global Initiative for Obstructive Lung Disease grading classification [56], which includes four stages, namely stage 1 (mild), stage 2 (moderate), stage 3 (severe) and stage 4 (very severe) [57]. Early-stage COPD includes stage 1 COPD and stage 2 COPD, which indicates that CS-induced COPD in mice only represents mild-to-moderate COPD in humans. Therefore, whether ADSCs-Exo can protect against severe-to-very severe COPD is still unknown. Moreover, the major factor involved in the protective effects of ADSCs-Exo remains unclear. Thus, further studies are still in urgent need to elucidate the protective roles of ADSCs-Exo in COPD caused by long-term CS exposure and in other COPD models. The treatment of ADSCs-Exo can inhibit the pyroptosis of AMs, however, the effect is modest. Considering the role of ADSCs-Exo in inhibiting apoptosis has been demonstrated [58], it would be very important to investigate the effect of ADSCs-Exo on the apoptosis of AMs. In addition, exploration of the main cargo responsible for the therapeutic effects of ADSCs-Exo would be another step forward.

## Conclusion

**Fig. 9:**
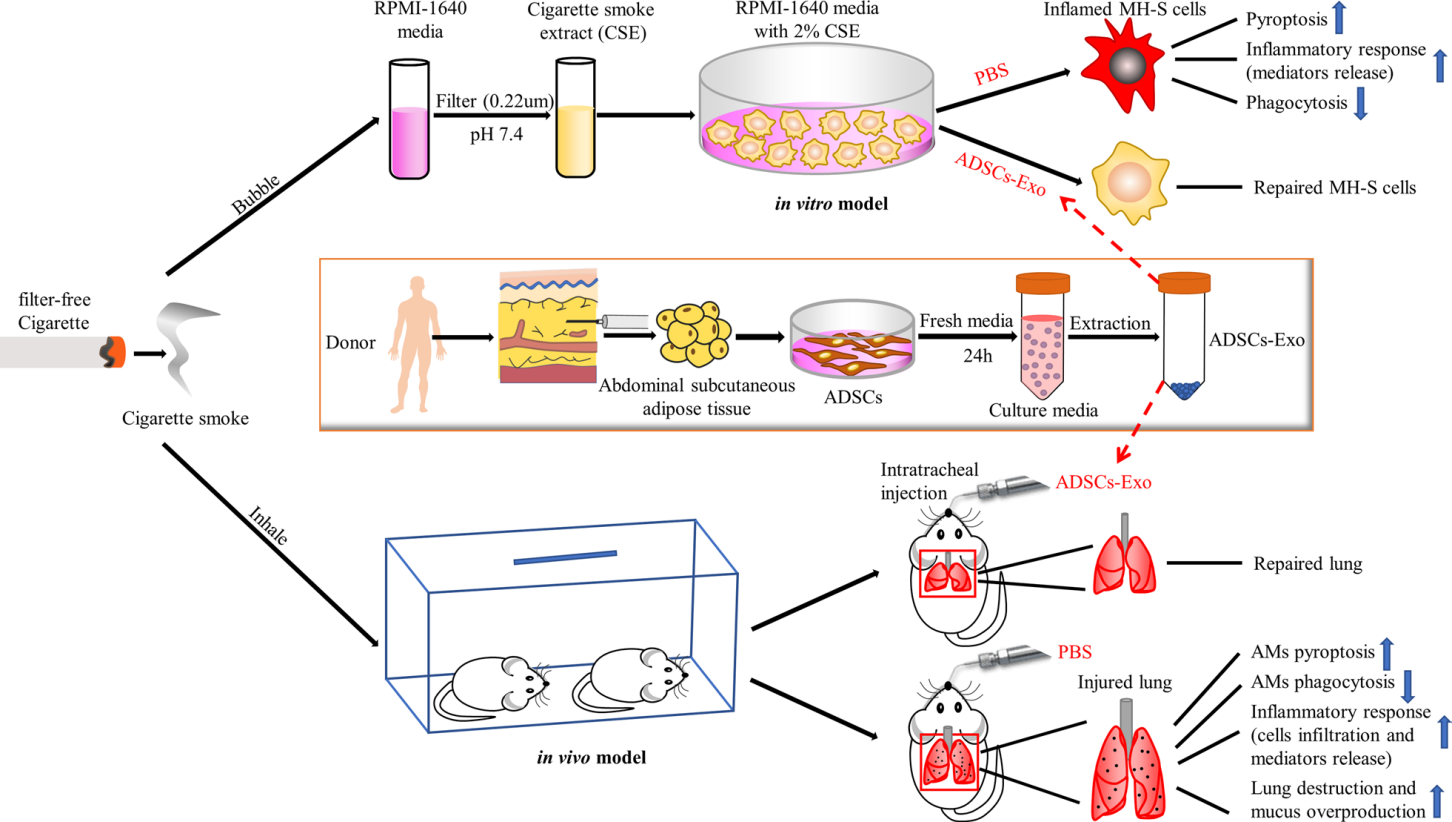
Graphical abstract. Graph showing that the administration of ADSCs-Exo ameliorated CS-induced lung inflammation, mucus overproduction, and injury as well as AMs pyroptosis

In summary, this research revealed that ADSCs-Exo is capable of mitigating lung inflammation and mucus hypersecretion caused by CS exposure, thereby attenuating CS-induced lung injury. In addition, this study also demonstrated that the potential involvement of AMs pyroptosis in CS-induced pulmonary inflammation and the inhibition of AMs pyroptosis potentially contributes to the protective roles of ADSCs-Exo in CS-induced lung inflammation and injury (Fig. 9). Collectively, ADSCs-Exo may represent a promising option for the treatment of lung inflammation and injury caused by CS.

## Supplementary Information

**Additional file 1: Figure S1.** Expression characteristic of hematopoietic markers in these primary culture cells. Flow cytometry shows that these cells were negative for CD34 (a) and CD45 (b).

## Data Availability

The datasets used and/or analyzed during the current study are available from the corresponding author on reasonable request.

## Abbreviations

COPD: Chronic obstructive pulmonary disease
CS: Cigarette smoke
MSCs: Mesenchymal stem cells
ADSCs: Adipose-derived MSCs
Exo: Exosomes
ADSCs-Exo: ADSCs-derived exosomes
AMs: Alveolar macrophages
MAMs: Mouse alveolar macrophages
AB: Alcian blue
PAS: Periodic acid Schiff
GSDMD: Gasdermin D
