# Supplementary figures and images for: Exosomes derived from adipose-derived stem cells alleviate cigarette smoke-induced lung inflammation and injury by inhibiting alveolar macrophages pyroptosis
Source: Respir Res. 2022 Jan 11;23:5. doi: 10.1186/s12931-022-01926-w (PMC8753876; doi:10.1186/s12931-022-01926-w)

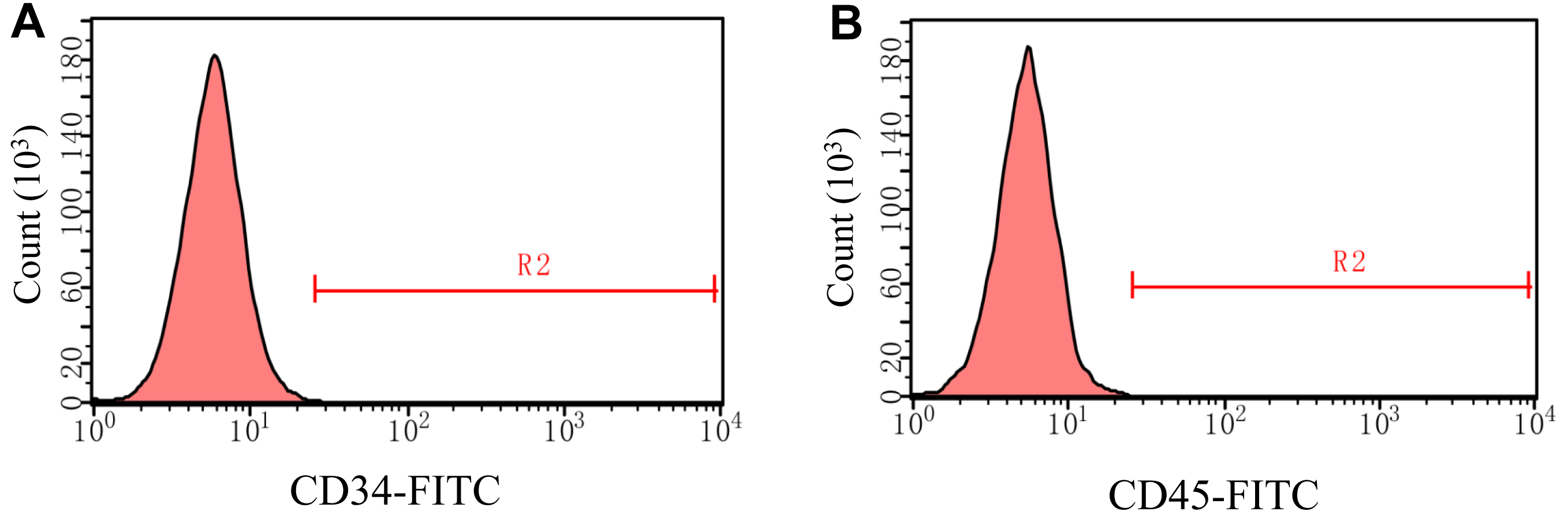

Supplement: Supplementary file 1 — Additional file 1: Figure S1. Expression characteristic of hematopoietic markers in these primary culture cells. Flow cytometry shows that these cells were negative for CD34 (a) and CD45 (b). [file 12931_2022_1926_MOESM1_ESM.tif]
